# Chorio-Epithelioma of Uterus

**Published:** 1912-09

**Authors:** 


CHORIO-EPITHELIOMA OF UTERUS, Michele Caturani, *Post Graduate,* Jan., 1912. The case was that of an Italian, aged 43, 8 children, 3 abortions. 15 months ago, after missing a menstruation, haemorrhages set in and the uterus was curetted. After several palliative operations, curettage, partial amputation of cervix, etc., the correct diagnosis was made by microscopic examination and a complete abdominal hysterectomy was performed in Jan., 1911, the patient remaining well to date.

